# Partially resistant *Cucurbita pepo* showed late onset of the *Zucchini yellow mosaic virus* infection due to rapid activation of defense mechanisms as compared to susceptible cultivar

**DOI:** 10.3389/fpls.2015.00263

**Published:** 2015-04-28

**Authors:** Slavomíra Nováková, Gabriela Flores-Ramírez, Miroslav Glasa, Maksym Danchenko, Roderik Fiala, Ludovit Skultety

**Affiliations:** ^1^Institute of Virology, Slovak Academy of SciencesBratislava, Slovakia; ^2^Institute of Botany, Slovak Academy of SciencesBratislava, Slovakia; ^3^Institute of Microbiology, Academy of Sciences of Czech RepublicPrague, Czech Republic

**Keywords:** *Cucurbita pepo* cultivars, *Zucchini yellow mosaic virus*, resistance to phytopatogen, plant biotic stress, oxidative stress, comparative proteomics, two-dimensional gel electrophoresis, mass spectrometry

## Abstract

*Zucchini yellow mosaic virus* (ZYMV) is an emerging viral pathogen in cucurbit-growing areas wordwide. Infection causes significant yield losses in several species of the family *Cucurbitaceae*. To identify proteins potentially involved with resistance toward infection by the severe ZYMV-H isolate, two *Cucurbita pepo* cultivars (Zelena susceptible and Jaguar partially resistant) were analyzed using a two-dimensional gel electrophoresis-based proteomic approach. Initial symptoms on leaves (clearing veins) developed 6–7 days post-inoculation (dpi) in the susceptible *C. pepo* cv. Zelena. In contrast, similar symptoms appeared on the leaves of partially resistant *C. pepo* cv. Jaguar only after 15 dpi. This finding was confirmed by immune-blot analysis which showed higher levels of viral proteins at 6 dpi in the susceptible cultivar. Leaf proteome analyses revealed 28 and 31 spots differentially abundant between cultivars at 6 and 15 dpi, respectively. The variance early in infection can be attributed to a rapid activation of proteins involved with redox homeostasis in the partially resistant cultivar. Changes in the proteome of the susceptible cultivar are related to the cytoskeleton and photosynthesis.

## Introduction

*Cucurbita pepo* (family *Cucurbitaceae*) is an important food plant cultivated worldwide. This species includes eight groups of edible cultivars (pumpkin, zucchini, scallops, acorns, crooknecks, straightnecks, vegetable marrows, and cocozelles) (Paris, 1989). Since the geographical barriers for pathogen movement have been reduced by globalization, international trade, and global climate changes (Mawassi and Gera, 2012), one of the most challenging tasks for sustainable food production is protection of crops against diseases. Phytopathogens (bacteria, viruses, fungi, nematodes, etc.) represent an increasing problem for agricultural productivity, because they negatively affect food quality and reduce yield.

*Zucchini yellow mosaic virus* (ZYMV, genus *Potyvirus*, family *Potyviridae*) is an emerging viral pathogen in the cucurbit-growing areas of tropical, subtropical, and temperate regions (Desbiez and Lecoq, 1997; Gal-On, 2007; Lecoq and Desbiez, 2008). Analogous to *Potato Virus Y* (Shand et al., 2009), it may induce cytopathic effect in plant cells, i.e., abnormal extension and transformation of mitochondrial structure, chloroplast anomalies such as accumulation of lipids and/or chloroplastic membranes changes. ZYMV has a relatively narrow host range beyond cultivated and wild cucurbits, infecting a few ornamental species (althea, begonia, delphinium) and weeds under natural condition (Lecoq and Desbiez, 2008). The virus is efficiently transmitted by more than 25 aphid species (Katis et al., 2006). Reports of seed-transmission are conflicting, and it is assumed that this is of low impact (Simmons et al., 2013).

ZYMV is responsible for vein clearing, mosaic, leaf deformation, and stunting in cucurbits, leading to complete yield loss if infection occurs early (Blua and Perring, 1989; Lecoq and Desbiez, 2012). The virus-host interaction is a complex biological phenomenon, that is affected by weather, season, viral isolate, and host susceptibility (Canto et al., 2009). The pathogen has to overcome various physical (cuticle, extracellular matrix) and chemical (secondary metabolites) barriers in order to penetrate the cells and induce non-specific (“non-host,” pattern-triggered immunity) and/or specific (“host,” effector-triggered immunity) plant defense responses. The latter is also referred to as gene-for-gene resistance, and is based on both direct and indirect interaction of nucleotide binding site-leucine rich repeat plant receptors (R-genes) (Bonardi et al., 2012) with their pathogenic elicitors (Avr-genes) (Nurnberger and Lipka, 2005; Jones and Dangl, 2006).

Because the majority of cultivated cucurbits manifest some form of resistance or tolerance to ZYMV (Desbiez and Lecoq, 1997), the course of infection and severity of the symptoms depend on specific interactions between the virus and the host cell components. This might involve changes in expression of hundreds of genes. Genetic analysis of *Cucurbita* cultivars identified several resistance-related candidates (Brown et al., 2003; Paris and Brown, 2005; Pachner et al., 2011). In *Cucurbita moschata* cross cv. Nigeria Local with cv. Waltham Butternut, three genes were found to be involved in resistance: the dominant gene *Zym-0* acts alone, and *Zym-4* has a complementary interaction with the recessive *zym-5*. Both *Zym-0* and *Zym-4* were also detected in cv. Nicklow's Delight, and the recessive *zym-6* is responsible for resistance in the related cv. Soler (Pachner et al., 2011). Finally, the major dominant *Zym-1*, and either of two complementary genes, *Zym-2* and *Zym-3* were responsible for resistance in *Cucurbita moschata* cv. Menina (Paris and Cohen, 2000).

**Figure 1:**
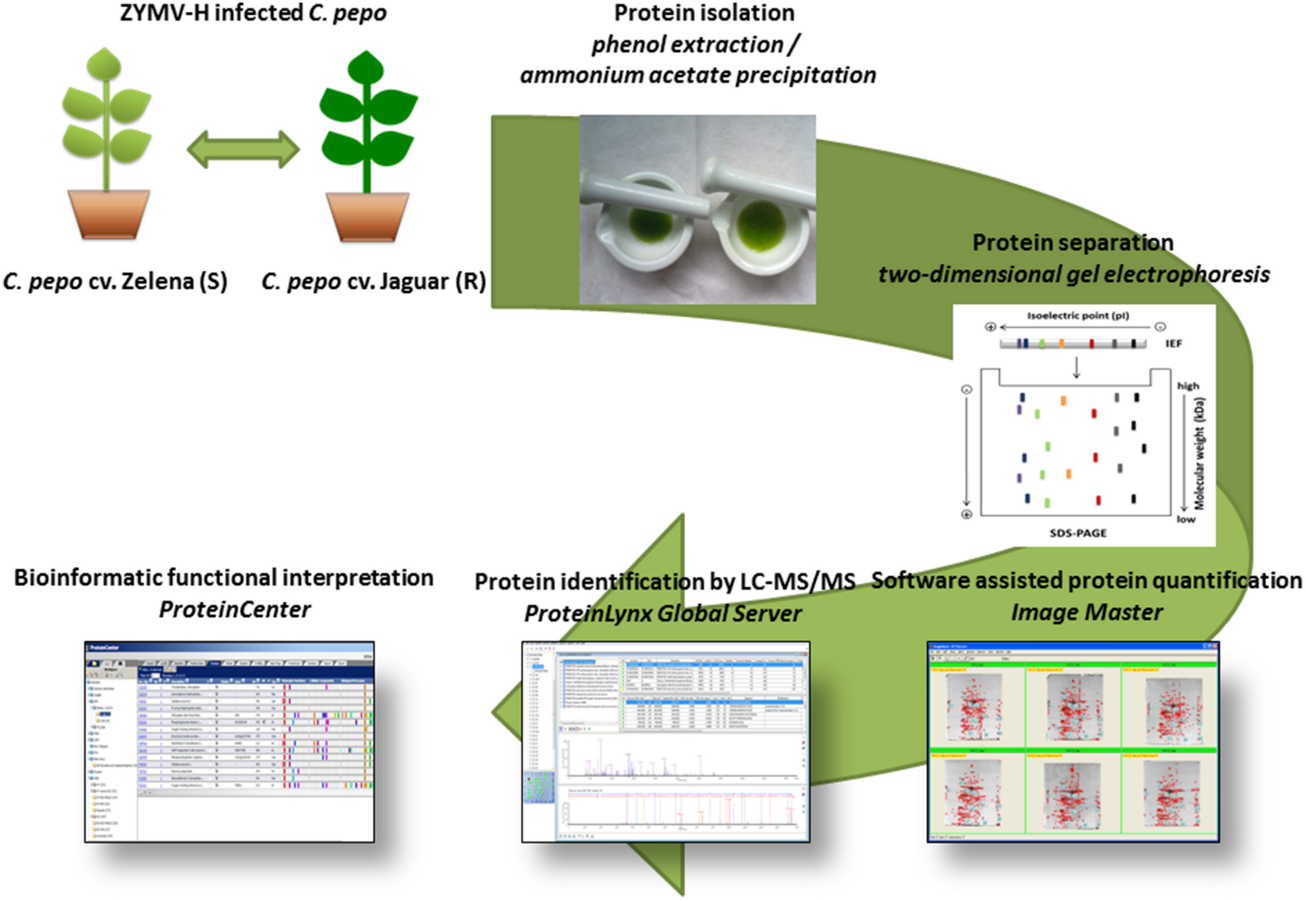
**Flow chart of the experimental design**.

In order to understand the molecular features that underlie the different performance of two *C. pepo* cultivars in response to infection with severe ZYMV-H isolate, we have employed a proteomic approach based on two-dimensional gel electrophoresis (2-DE) combined with liquid chromatography coupled tandem mass spectrometry (LC-MS/MS) identification (Figure 1). We chose this omics-based strategy not only to provide a global perspective of extraordinary intricacy of mechanisms with which a simple viral genome perturbs the plant cell molecular networks of the cultivars, but also to reveal protein targets/markers useful in the design of future diagnosis and/or plant protection strategies.

## Materials and methods

### Plant growth and virus infection

Two cultivars of zucchini, *C. pepo* Zelena (referred to as susceptible) and Jaguar (referred to as partially resistant) were used in this study. Plants were grown in a growth chamber under controlled conditions (14 h light/10 h dark photoperiod, 55 μmol m^−2^s^−1^ photon flux density, day/night temperature: 25/18°C). Carborundum-dusted cotyledons of both cultivars were mechanically inoculated with the same dose of ZYMV (severe isolate H (ZYMV-H) UniGene accession number KF976712) (Glasa et al., 2007) at the 2 true-leaf seedling stage (~14 days after sowing). Development of symptoms was evaluated visually at 6 and 15 days post-inoculation (dpi).

### Confocal laser scanning microscopy

2′,7′-dichlorodihydrofluorescein diacetate (H2DCFDA) was used as an indicator for H_2_O_2_ accumulation in cells. Leaves were stained 15 min with 50 μM H2DCFDA in 50 mM phosphate buffer pH 7.5, washed for 2 min in distilled water and observed in confocal microscope Olympus FV1000 (Olympus, Japan). The excitation wavelength was 488 nm and fluorescence was detected using emission barrier filter 505–550 nm.

### Protein extraction and quantification

Proteins were extracted from young leaves of both cultivars of infected plants harvested separately at 6 and 15 dpi. In all cases, 1 g (fresh weight) of leaves was ground to a fine powder in liquid nitrogen using a mortar and pestle. The powder was subjected to a phenol extraction/ammonium acetate precipitation protocol (Klubicova et al., 2011). Briefly, homogenization buffer [50% (w/v) phenol, 0.2% (v/v) 2-mercaptoethanol, 50 mM Tris-HCl, pH 8.8, 5 mM EDTA, and 450 mM sucrose] was added to the powder. After 30 min at 4°C, samples were clarified by centrifugation. Proteins from the upper phenol phase were precipitated overnight with 0.1 M ammonium acetate in methanol and washed consecutively with 0.1 M ammonium acetate in methanol, 80% acetone and 70% ethanol. Protein concentration was determined by the method of Bradford (Bradford, 1976). Samples were then divided into aliquots containing 750 μg of protein. For prolonged storage, precipitated proteins were kept in 70% (v/v) ethanol at −80°C.

### SDS PAGE and western blot analysis

Immunoblot analyses were used to determine the time course of virus accumulation in both cultivars. Briefly, protein samples were dissolved in a solution containing 8% (w/v) SDS, 240 mM Tris, pH 6.8, 40% (v/v) glycerol, 2% (v/v) 2-mercaptoethanol, and 0.04% (v/v) Bromphenol blue. Then, electrophoresis was carried out using 12.5% acrylamide gels (8.3 cm × 7.3 cm × 0.75 mm) with Tris-glycine running buffer, pH 8.3 using Mini-Protean (Bio-Rad, USA) apparatus. Separations were performed at 60V for 15 min, followed by 200V until tracking dye reached the bottom of the gels. The separated proteins were transferred to a PVDF membrane (Schleicher Schuell, Germany) using a semi-dry blotting apparatus (Bio-Rad, USA). The quality of protein transfer was evaluated by staining the membranes with 0.1% (w/v) Ponceau S (Sigma Aldrich, USA) in 5% (v/v) acetic acid. Membranes blocked with 5% (w/v) fat-free milk were then incubated overnight with 1000x diluted rabbit polyclonal antibody to ZYMV coat protein (BIOREBA, Switzerland) at 4°C. Membranes were washed three times for 10 min in phosphate saline buffer (PBS) and incubated with alkaline phosphatase-conjugated goat anti-rabbit IgG (Sigma Aldrich, USA) in the dark for 3 h at room temperature (RT). Colorimetric detection was achieved by adding a mixture of nitro blue tetrazolium chloride and 5-bromo-4-chloro-3-indolyl phosphate (Thermo Scientific, Germany/Serva, Germany) in the ratio 2:1 (v/v) dissolved in a buffer containing 0.1 M Tris, pH 9.0, 0.1 M NaCl, and 10 mM MgCl_2_.

### 2-DE separation and image analysis

The precipitated proteins were dissolved in a sample buffer (8M urea, 2M thiourea, 2% (w/v) CHAPS, 2% (v/v) Triton X-100, 50 mM DTT) at RT for an hour with gentle agitation. Insoluble material was removed by centrifugation at 14,000 g for 20 min at RT. Carrier ampholytes (pH 3-10) were added to the soluble samples to a final concentration of 1% (v/v). Immobilized pH gradient strips (pH 3-10, 18 cm, GE Healthcare, Sweden) were passively rehydrated overnight (~16 h) in the dark at RT, then placed into Multiphor II apparatus (GE Healthcare, Sweden) and isoelectric focusing was performed using the following protocol: 150 V for 1.5 h, 200 V for 1.5 h, 600 V for 2 h, 1000 V for 2 h, 1500 V for 2 h and 5000 V for 15 h. The strips were then rinsed in deionized water and incubated in equilibration buffer (50 mM Tris-HCl, pH 8.8, 6M urea, 30% (v/v) glycerol, 2% (w/v) SDS containing 1% (w/v) DTT) for 15 min, followed by 4% (w/v) iodoacetamide with 0.08% (w/v) Bromphenol blue. The second dimension separation was carried out on 12.5% polyacrylamide gels (20 cm × 20 cm × 1 mm) in Tris-glycine running buffer, pH 8.3, using a Protean XL (Bio-Rad, USA) device. It was performed at 5 mA/gel for 90 min, followed by 45 mA/gel until tracking dye migrated to the bottom of the gels. Protein spots were visualized with colloidal Coomassie Brilliant Blue. After destaining with deionized water, the gels were scanned at 300 dots per inch and 16-bit grayscale. Triplicate gel images of both groups (Figure S1) were quantitatively analyzed using ImageMaster 2D Platinum 4.9 software (GE Healthcare, Sweden). Automatic spot detection was performed with the following settings: Smooth 2 (software smoothes the image 2 times to optimize splitting of overlapping spots); Saliency 1 (parameter based on spot curvature to filter the noise); Min. area 5 pixels (to filter intense dust particles). Statistical significance of differences was assessed by the Student's *t*-test. Only the spots presented in at least two gels out of the three replicates which showed a significant difference (*p* ≤ 0.05) and fold change ≥1.5 were manually excised from the gels and analyzed further.

### In-gel trypsin digestion

The excised protein spots were washed with agitation in 50% (v/v) acetonitrile (ACN) (Merck, Germany) in 50 mM NH_4_HCO_3_(ABC; Fluka, Switzerland) at RT. After complete destaining, gel pieces were dehydrated with 100% ACN for 10 min at RT, reduced in 15 mM DTT, and alkylated in 30 mM iodoacetamide. The gel plugs were washed again with 50 mM ABC for 10 min at RT (3x), dehydrated with 100% ACN, and then incubated for 14–16 h at 37°C in digestion solution (40 ng of lyophilized sequencing grade modified trypsin (Promega, USA) per 5 μL of 50 mM ABC. The resulting peptides were acidified in extraction solution (1% (v/v) formic acid (FA; Sigma Aldrich, USA) in 5% (v/v) ACN) followed by dehydration of the gel pieces in 50% ACN. Total volume of the samples was reduced to 20 μl by vacuum evaporation and samples were stored at −20°C until LC-MS/MS.

### Mass spectrometry

The tryptic peptides were analyzed by automated nanoflow reverse-phase chromatography using the nanoAcquity UPLC system coupled to a Q-TOF Premier (Waters, USA) as described earlier (Skultety et al., 2011; Jankovicova et al., 2013; Flores-Ramirez et al., 2014). Peptides were injected onto a reverse-phase column (Waters nanoAcquity UPLC column BEH 130 C18, 75 μm × 150 mm, 1.7 μm particle size). An ACN gradient (6–40% B in 15 min; A = water with 0.1% (v/v) FA, B = ACN containing 0.1% FA) at a flow rate of 350 nL/min was used to elute peptides into the tandem mass spectrometer. The column was directly connected to the PicoTip emitter (New Objective, USA) mounted into the nanospray source. A nano-electrospray voltage of 3.5 kV was applied, with the source temperature set to 70°C. Data were acquired by a multiplex approach called MSE (Uvackova et al., 2013, 2014) using alternate scans at low and high collision energies. Spectral acquisition scan rate was 0.8 s, with a 0.05 s inter-scan delay. In MS channel, data were collected at constant collision energy 4 eV and the fragments were recorded while collision energy ramped from 20 to 35 eV in MS/MS channel. Ions with 100–1900 m/z were detected in both channels, however, the quadrupole mass profile settings allowed efficient deflection of masses less than 400 m/z in low energy mode to filter out contaminating ions.

### Protein identification and functional interpretation

The data were processed using the ProteinLynx Global Server (PLGS) v. 3.0 (Waters, UK) that provideded noise-filtering at the following threshold parameters: low energy 60 counts, high energy 150 counts, intensity 1200 counts. In order to produce a single accurate monoisotopic mass for each peptide and the associated fragment ions, the deisotoped, lockmass-corrected, and centroided data were charge-state reduced. The time alignment was used to initially correlate the precursor and fragment ions. All data were lockspray calibrated against [Glu1]-Fibrinopeptide B (Sigma Aldrich, USA). The results were searched against the Cucurbitaceae sequences downloaded from NCBI (http://www.ncbi.nlm.nih.gov/protein/ in June 2014 containing 51,869 entries) and a Swissprot database (http://www.uniprot.org/ containing 545,536 entries downloaded in June 2014). The algorithm also incorporates a random decoy database to determine the false-positive identification rate that was set as acceptable up to 4%. During database searches, one missed cleavage site was allowed. The precursor peptide mass tolerance was set to ±15 ppm, and fragment mass tolerance to ±40 ppm. The search was performed with Cys carbamidomethylation and Met oxidation as fixed and variable modifications, respectively. A minimum of two matched peptides and three or more consecutive fragment ions from the same series were required for protein identification. Protein identifications were accepted after manual inspection of probabilistic based PLGS assignment at 95% confidence level. Only those proteins are listed in the tables which were found at least twice out of three biological replicates. Functional interpretation of the findings was facilitated by bioinformatic study including data processing by ProteinCenter v. 3.12 (Thermo Scientific, Germany).

## Results

### Partially resistant *C. pepo* cultivar showed late onset of infection and lower viral accumulation at early stage

**Figure 2:**
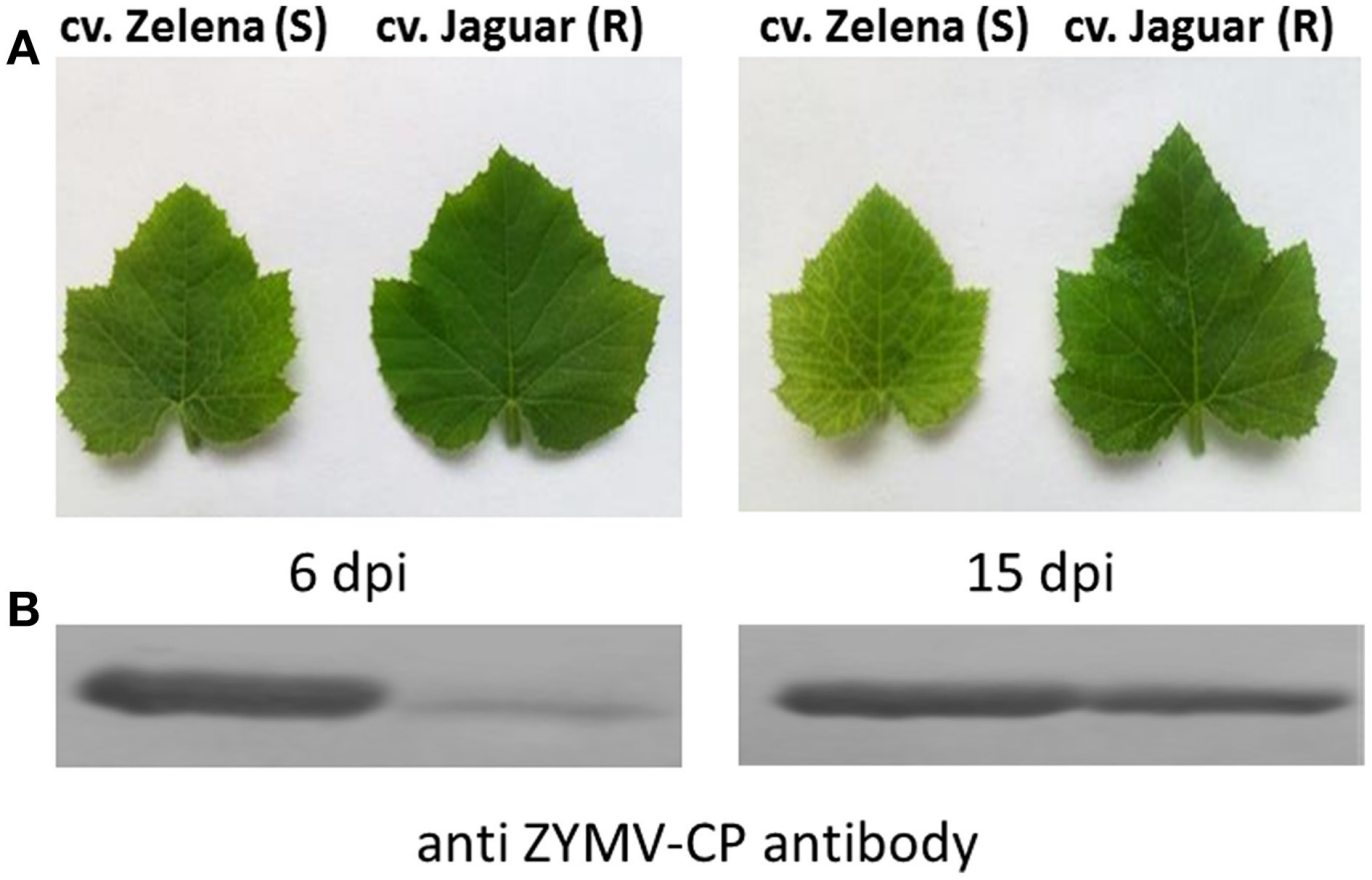
**Observed phenotypic symptoms (A) and growth of ZYMV on leaves of two *C. pepo* cultivars (cv. Zelena-susceptible (S) and cv.Jaguar partially resistant (R)) by immunoblot analysis (B)**. Protein load was normalized to RuBisCO.

In order to evaluate pathological effects of viral infection on leaves and assess their resistance, two cultivars of *C. pepo* (Zelena-susceptible and Jaguar-partially resistant) were inoculated with the severe ZYMV-H isolate. Initial symptoms on leaves (clearing veins) were developed 6–7 days after inoculation in the susceptible *C. pepo* cv. Zelena. In contrast, similar symptoms appeared on the leaves of partially resistant *C. pepo* cv. Jaguar only after 15 dpi. At this point, generalized systemic leaf chlorosis was observed on susceptible cultivar (Figure 2). This pattern was reproducible, as shown by 3 independent inoculation experiments.

**Figure 3:**
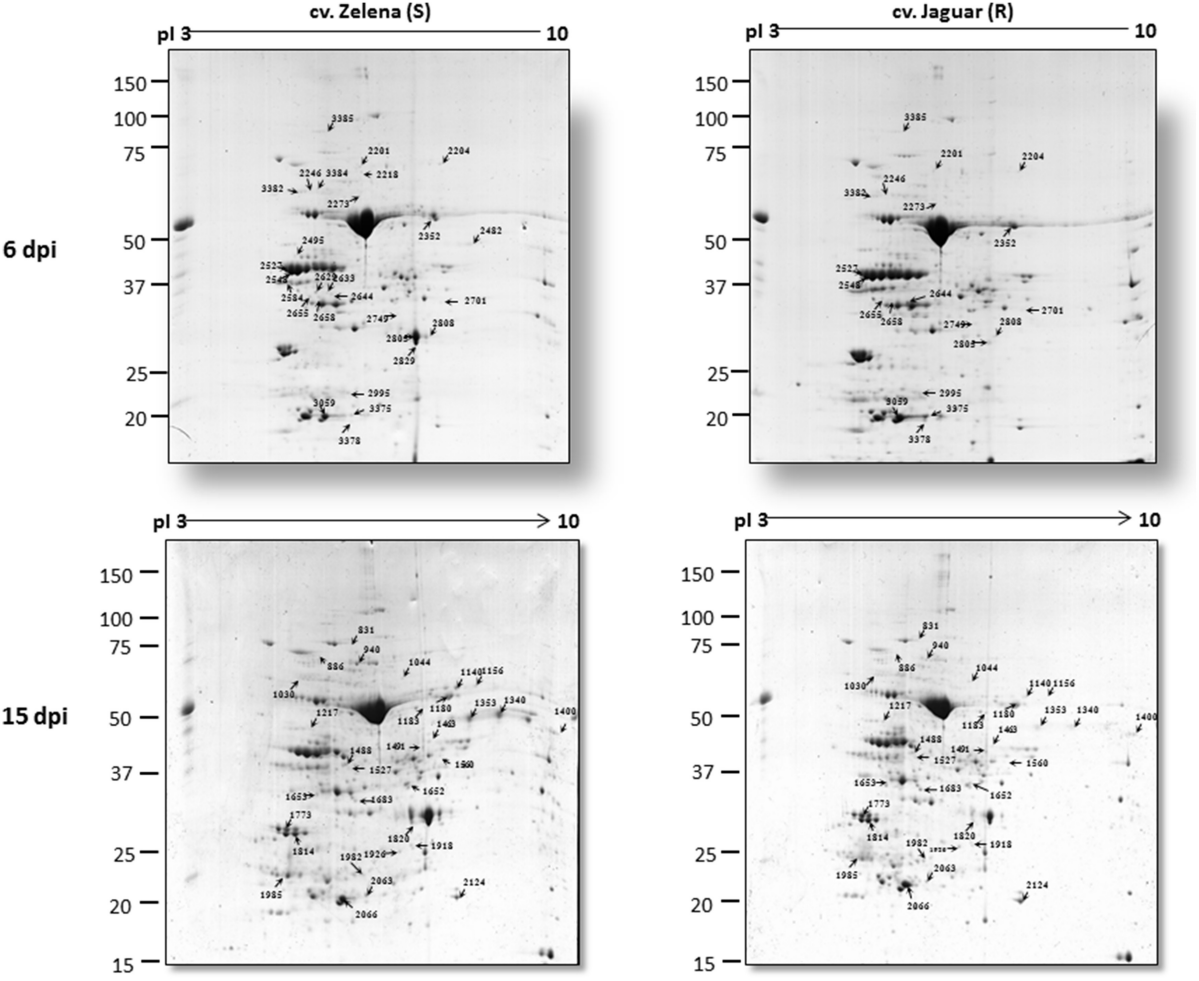
**Representative 2-DE gels of Zelena (S) and Jaguar (R) *C. pepo* cultivars at 6 and 15 dpi with ZYMV**. Arrows indicate the positions of differentially displayed protein spots.

Immunoblot analysis detected almost 50 times higher viral accumulation at 6 dpi in cv. Zelena as compared to cv. Jaguar (Figure 2). In contrast, the abundance of the virus was comparable at the later stage of infection (15 dpi). Our proteomics-based observations are in good agreement with this result. A significantly (18–35 times) higher level of ZYMV-H coat protein was detected in Zelena cultivar compared to Jaguar at 6 dpi (spots 2805, 2808, and 2829). However, only an order of magnitude lower difference in abundance was observed at 15 dpi (spot 1820) (Figure 3). A similar correlation was observed with the ZYMV P3N-PIPO polyprotein, which was found to be highly overrepresented in susceptible cultivar at 6 dpi (spot 2482) but only about 5 times at 15 dpi (spots 1340 and 1353).

### Profiling of the leaf proteome reveals differential response of *C. pepo* cultivars to ZYMV-H infection

In order to reveal changes in protein composition because of ZYMV-H infection, profiling of the leaf proteome was conducted using 2-DE. Total proteins were resolved within the pI range 3–10 and mass range 15–150 kDa. The analyses were performed on samples collected in three biological replicates for both cultivars at 6 (early stage of the response to infection) and 15 (disease development) dpi (Figure S1). Representative gels are shown in Figure 3. It was apparent that many of the protein spots were common to both cultivars. Image analysis revealed 371 and 440 pairs of protein spots which were reproducibly detected on CBB-stained gels at 6 and 15 dpi, respectively. Inspection of the abundance ratio of corresponding protein spots indicated that over 100 of them were differentially abundant. However, the protein spots were considered significantly different only if (i) similar difference in abundance was observed between the *C. pepo* cultivars in at least two out of three biological replicates and (ii) if the average intensity of protein spots altered by at least 1.5-fold (*p* ≤ 0.05). In summary, we identified 59 substantially different protein spots (Figures S2, S3); 28 were detected at 6 dpi and 31 at 15 dpi. Enlarged views of the corresponding electrophoretic patterns and a bar chart expressing quantitative changes are shown in Figures S2, S3, respectively.

### ZYMV-H infection of *C. pepo* cultivars affected mainly chloroplastic proteins involved in photosynthesis and defense mechanisms

Differentially abundant protein spots were excised from the gels, digested with trypsin, and analyzed by LC-MS/MS. Subsequent data were processed by PLGS and experimentally recorded MS spectra were matched against *Cucurbitaceae* and SwissProt databases. Likely due to lack of fully-sequenced genome of *C. pepo*, as many as 17 gel spots contained proteins that could not be identified. Specifically, 23 and 19 proteins were identified at 6 and 15 dpi (Table 1, Table S1), respectively. The photosystem II stability/assembly factor HCF136 (2633, 2629), ribulose bisphosphate carboxylase/oxygenase activase 1 (2527, 2548) and RuBisCO-binding protein subunit beta (3382, 2246) were found in two spots at 6 dpi. Likewise, oxygen evolving enhancer protein 1 (1814, 1773) was found in two spots at 15 dpi. Further examination of electrophoretic patterns of these proteins revealed only slight differences in their pIs and/or MWs, likely due to post-translational modifications. For example, the ribulose bisphosphate carboxylase/oxygenase activase 1 spots appeared on the gels as a “string of pearls” which is typical for the proteins with multiple phosphorylation states. Among the 23 protein spots identified at 6 dpi, 18 showed higher accumulation in cv. Zelena (susceptible) as compared to cv. Jaguar (partially resistant). Although, this proportion has changed at 15 dpi, where majority (10) of 19 identified proteins was more abundant in partially resistant cultivar.

**Table 1:**
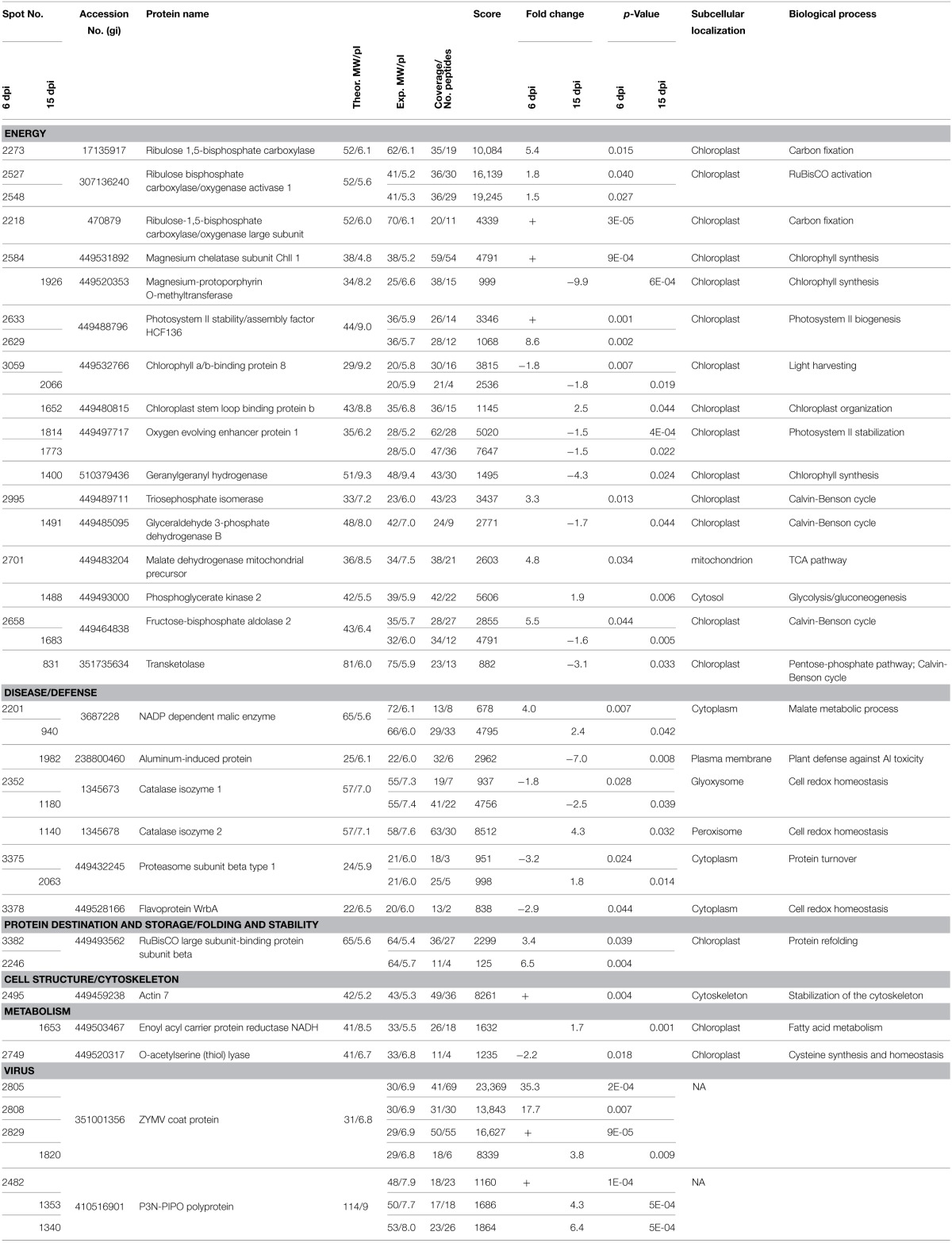
**Proteins identified by LC-MS/MS analysis that were significantly changed between *C. pepo* cultivars at 6 dpi and 15 dpi**.

*Alterations in protein abundance are related to susceptible cultivar*.

**Figure 4:**
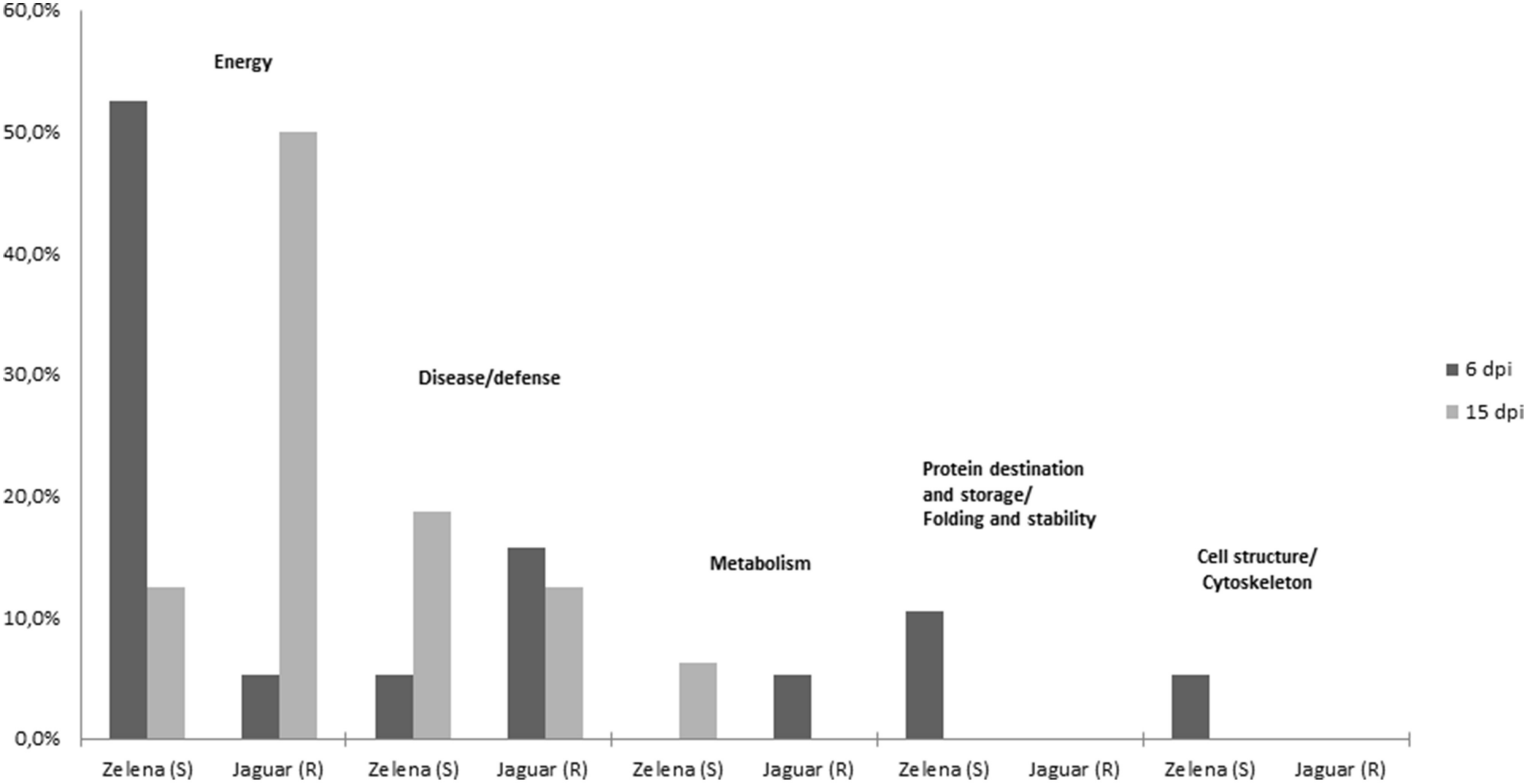
**Functional categories of differentially abundant proteins**.

**Figure 5:**
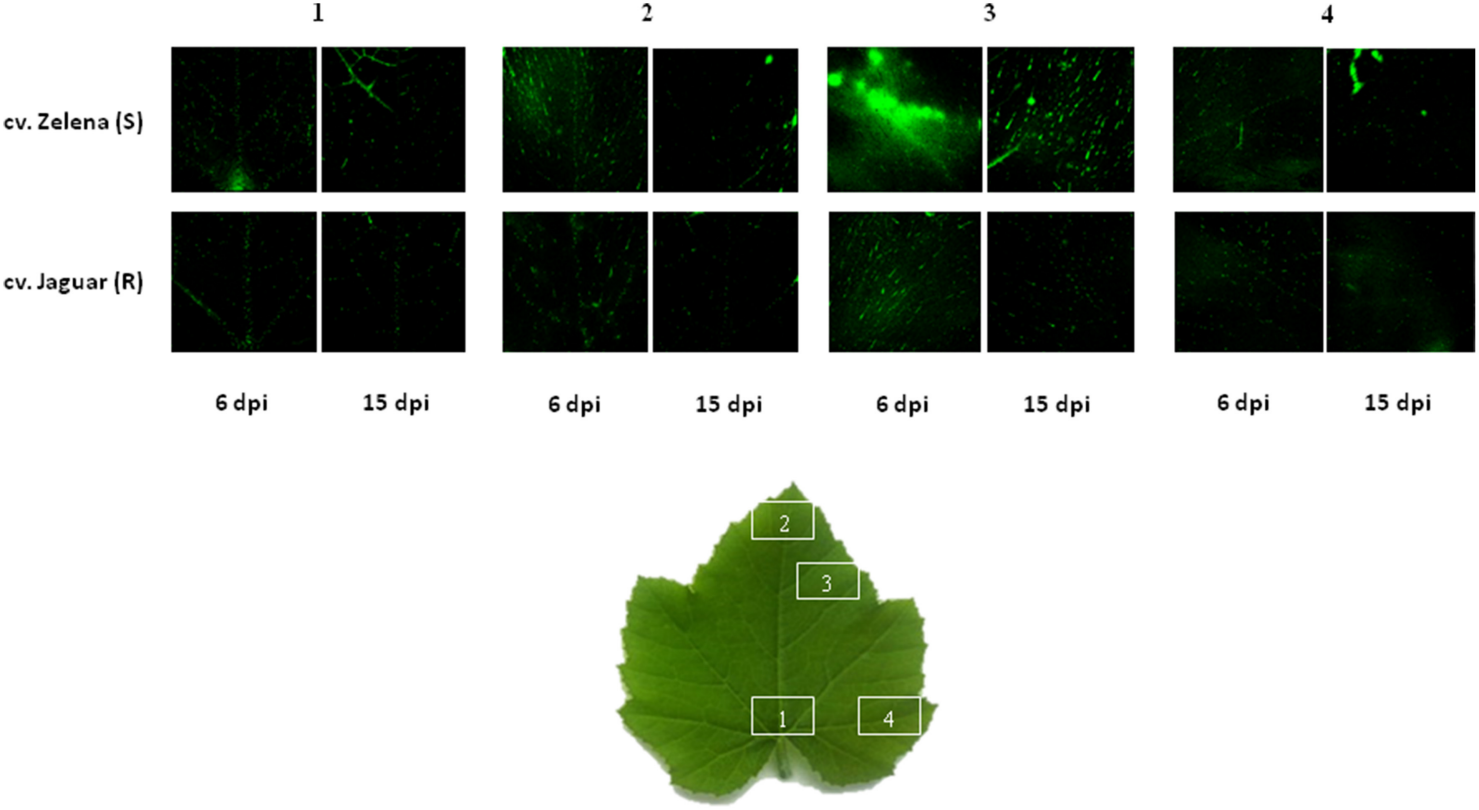
**Confocal microscopy micrographs of the hydrogen peroxide production detected by the fluorescent probe in the leaves of two *C. pepo* cultivars cv. Zelena-susceptible (S) and cv. Jaguar partially resistant (R) at 6 and 15 dpi infected with severe ZYMV-H**. H_2_O_2_ production is indicated by green fluorescence.

Although, we understand that these proteins are not necessary involved in resistance of *C. pepo* cultivars toward infection by the severe ZYMV-H isolate, we believe that due to strict protocol, most of the 26 identified plant proteins (Table 1) exhibited reproducible and significant changes under this influence. These identified responsive proteins were clustered into five functional categories according to classification model developed for plants (Bevan et al., 1998), including proteins associated with photosynthesis and other energy (carbohydrate) metabolism, defense against stress, protein destination and storage, common metabolic pathways, and cell structure maintenance (Table 1, Figure 4). The gel spots containing proteins implicated in photosynthesis, carbohydrate metabolism, and defense comprise 79 and 94% of the proteins identified at 6 and 15 dpi, respectively. Involvement of proteins associated with detoxification of reactive oxygen species (ROS) was evaluated by hydrogen peroxide analysis (Figure 5).

## Discussion

The plant-virus interaction is a complex pathophysiological phenomenon, in which resistance, defense, susceptibility, and direct virus-induced reactions interplay to trigger expression responses of hundreds of gene products. Proteins associated with the photosynthetic apparatus, energy metabolism/protein synthesis and turnover are typically involved. Modulation of metabolism related to sugars, cell wall, ROS or pathogenesis has been previously reported as well (Babu et al., 2008; Yang et al., 2011; Di Carli et al., 2012; Figueiredo et al., 2012; Petriccione et al., 2013; Wu et al., 2013a,b). Although, most of these studies examined the plant-pathogen interactions, they did not paid attention to biological properties of different cultivars. Thus, our initial proteomics-based analysis explored different performance of two *C. pepo* cultivars: Zelena (susceptible) and Jaguar (partially resistant) in response to infection with severe ZYMV-H isolate.

### Alterations in abundance of specific proteins suggest an activation of repairing mechanisms for preservation of photosynthesis in *C. pepo* cultivars

Most of the identified differentially displayed proteins were associated with photosynthesis and photorespiration at the both periods. They were more abundant in cv. Zelena during the early stage of infection (at 6 dpi) and accumulated in cv. Jaguar at 15 dpi. In the susceptible cultivar, all photosynthetic enzymes beside chlorophyll a-b binding protein (spot 3059, almost 2 times less accumulated) were more abundant during the early stage of infection (6 dpi). Among these enzymes, is RuBisCO, the key enzyme of photosynthesis catalyzing oxygenation (in photorespiration) or carboxylase reaction (in the Calvin-Benson cycle) of ribulose 1,5-bisphosphate into 2-phosphoglycolate and/or 3-phosphoglycerate (Peterhansel et al., 2010). Its activation is ensured by the presence of RuBisCO activase. Two additional enzymes, a chloroplastic isoform of triosephosphate isomerase (spot 2995, >3 times more accumulated in susceptible cultivar), and fructose-bisphosphate aldolase (spot 2658, >5 times more), are components of the Calvin-Benson cycle. From other chloroplast proteins, photosystem II (PSII) stability/assembly factor HCF136 (spots 2633 and 2629, >9 times more) and magnesium chelatase subunit ChlI1 (spot 2584) were found as highly abundant at early stage of infection. Magnesium chelatase catalyzes the insertion of Mg^2+^ into protoporphyrin IX, the first step downstream from the branchpoint of chlorophyll biosynthesis (Kobayashi et al., 2008) that is a highly regulated process. The corresponding product, Mg protoporphyrin IX methyl ester, has been proposed to play an important role as a signaling molecule implicated in plastid-to-nucleus communication (Pontier et al., 2007). The PSII stability/assembly factor is essential for assembly of early intermediate of PSII (Plucken et al., 2002) that is a protein-pigment complex of light phase of photosynthesis catalyzing electron transfer from water to plastoquinone (Peng et al., 2006).

At later stage (15 dpi) only phosphoglycerate kinase 2 (spot 1488, almost 2 times more) catalyzing the reversible transfer of a phosphate group from 1,3-bisphosphoglycerate to ADP and chloroplast stem loop binding protein b (spot 1652, >2 times more) were confirmed in the susceptible cultivar as more accumulated. The later enzyme is an RNA-binding protein associated with structural integrity of chloroplast and defense response (Jones and Dangl, 2006; Bollenbach et al., 2009; Qi et al., 2012) that is usually highly expressed in seedlings and young leaves. All the other proteins associated with these activities were more abundant in the partially resistant cultivar at this period. In addition to transketolase (spot 831, >3 times more), fructose-bisphosphate aldolase (spot 1683, almost 2 times more), and glyceraldehyde 3-phosphate dehydrogenase (spot 1491, almost 2 times more) that are associated with Calvin-Benson cycle, there were identified two proteins, the geranylgeranyl hydrogenase (spot 1400, >4 times more) and magnesium-protoporphyrin O-methyltransferase (spot 1926, almost 10 times more), related to chlorophyll biosynthesis. It was demonstrated (Pontier et al., 2007) that chlorophyll formation is totally dependent on the magnesium-protoporphyrin IX methyltransferase in *Arabidopsis*. This enzyme catalyzes the transfer of the methyl group from S-adenosyl-L-methionine to magnesium-protoporphyrin IX to form magnesium protoporphyrin methyl ester. Inactivation of its gene prevents setting up of chlorophyll-binding proteins which subsequently affect the photosystem I and II and cytochrome b6f complexes. The both claims were confirmed by accumulation of additional enzymes involved in these processes, the oxygen-evolving enhancer protein 1 (spots 1814 and 1773, almost 2 times more) and the chlorophyll a/b-binding protein 8 (spot 2066, almost 2 times more). The first is a nuclear-encoded chloroplast protein peripherally bound to PSII on the lumenal side of the thylakoid membrane that is an essential for oxygen evolving complex activity and PSII stability (Robinson and Klosgen, 1994; Suorsa and Aro, 2007). The other is a component of the light-harvesting complex that plays an important role in regulation of the amount of absorbed light energy and thereby electron flow between photosystems I and II (Jansson, 1994). It was reported that its gene expression is down-regulated by high-light stress (Staneloni et al., 2008) because photoreduction of molecular oxygen by PSII may generate a superoxide anion radical (Pospisil, 2009).

This result agrees well with the symptoms observed on the plant leaves (Figure 2), and with the results from previous studies (Babu et al., 2008; Yang et al., 2011; Di Carli et al., 2012; Figueiredo et al., 2012; Petriccione et al., 2013; Wu et al., 2013a,b). For example, in shoots of susceptible cultivar of *Actinidia chinensis*, increased photosynthetic activity was observed during bacterial infection (Petriccione et al., 2013). Similar result has been reported by study of soybean-*Soybean mosaic virus* (SMV) interaction from transcriptomic point of view (Babu et al., 2008). Infected compared to healthy plant of soybean [G. max (L.) Merr.] “Williams 82” (susceptible cultivar) showed alterations in transcripts encoding proteins for chloroplast function and photosynthesis. They were accumulated at early (7 dpi) and downregulated at later stage (14 dpi) of infection. Whereas, the resistant cultivar of soybean “Kefeng No.1” showed downregulation of many photosynthetic proteins at 48 h post-inoculation with this virus (Yang et al., 2011). Interestingly, the concentration of proteins related to photosynthesis was decreased also in susceptible and resistant cultivars of maize during infection with *Sugarcane mosaic virus* (SCMV) at 6 dpi (Wu et al., 2013a). The difference was more apparent at 14 dpi (Wu et al., 2013b). At this time point the photosynthesis related proteins were >3 times more abundant in susceptible compared to resistant cultivar. As we observed higher viral accumulation and increased abundance of proteins associated with photosynthesis and photorespiration at 6 dpi in susceptible and later (15 dpi) (Figure 2) in the partially resistant cultivars, we conclude that the plant activates repairing mechanism for preservation of photosynthesis.

### Accumulation of proteins involved in detoxification of ROS at early stage of infection mainly in partially resistant cultivar indicates hypersensitive response to pathogen attack

A major hallmark of plant defense is the increased production of ROS, such as singlet oxygen (^1^O_2_), hydrogen peroxide (H_2_O_2_), superoxide (O^−^_2_), and hydroxyl radicles (HO^*^) (Alvarez et al., 1998). Although, these species are damaging at high concentrations, they are involved in signaling reactions that contribute to activation of defense responses at low concentrations (Apel and Hirt, 2004; Gadjev et al., 2006). Photosynthetic organisms have developed extensive antioxidant networks and redox buffering systems in order to prevent damaging cellular oxidation and to maintain redox homeostasis (Vranova et al., 2002). An accumulation of stress and defense-related proteins was observed at 48 h post-inoculation with SMV in resistant Kefeng No.1 (Yang et al., 2011) and later (14 dpi) in susceptible William 82 (Babu et al., 2008) soybean cultivars. Likewise, increased concentration of these proteins was also detected in the both susceptible and resistant cultivars of maize infected by SCMV at 6 and 14 dpi (Wu et al., 2013a,b). Thus, we hypothesized that the increased levels of defense-related proteins which occur at the early stage of infection (6 dpi) in the partially resistant cultivar Jaguar might be one of the main reasons for lower accumulation of the virus in infected leaves. This postulation agree well with the images of peroxide fluorescence on leaves of two *C. pepo* cultivars at 6 and 15 dpi (Figure 5). At early stage of infection (6 dpi), higher fluorescence of H_2_O_2_ was observed only in susceptible cultivar. Thus, we can speculate that it could be related with higher accumulation of proteins involved in detoxification of ROS in partially resistant and at later time point in the both cultivars.

We identified five enzymes associated with defense in this study. The isoforms of catalase that decompose hydrogen peroxide to water and oxygen, were differentially abundant in three spots (isoenzyme 1 in spots 2352 and 1180 and isoenzyme 2 in spot 1140). While isoenzyme 1 was detected at both time points post-infection as more abundant in partially resistant cultivar (1.8 and 2.5 times at 6 and 15 dpi, respectively), isoenzyme 2 was accumulated over 4 times in susceptible cultivar at 15 dpi only. In contrast, the flavoprotein WrbA (quinone oxidoreductase) that catalyzes reduction of quinones to dihydroquinones was identified only in partially resistant cultivar at 6 dpi (spot 3378, almost 3 times more accumulated). This enzyme might prevent formation of semiquinones, unstable and reactive intermediates which can lead to generation of ROS (Heyno et al., 2013). Similarly, the chloroplastic O-acetylserine(thiol)lyase (spot 2749) was found to be more abundant (>2 times more) only at 6 dpi in the partially resistant cultivar. It catalizes the formation of cysteine from O-acetylserine and hydrogen sulfide, which is likely involved in cysteine homeostasis and also in the global regulation of S-assimilation in plants. An isoform of this enzyme is essential for light-dependent redox regulation within the chloroplast due to sensing the redox status. It detects the accumulation of thiosulfate resulting from inadequate detoxification of ROS and forms S-sulfocysteine, which triggers protection mechanisms of the photosynthetic apparatus (Gotor and Romero, 2013). In addition, cysteine is required for synthesis of glutathione and methionine, the metabolic precursor S-adenosyl-L-methionine which is a major methyl-group donor in transmethylation reaction as well as intermediate in the biosynthesis of phytohormone ethylene, associated with modulation of plant response to stress (Ravanel et al., 1998).

Furthermore, a relationship between the cytoplasmic isoform of NADP-dependent malic enzyme and defense response has been reported (Maurino et al., 2001; Doubnerova et al., 2009). This enzyme catalyzes the oxidative decarboxylation of L-malate (in the presence of Mg^2+^ or Mn^2+^ ions) to produce pyruvate, CO_2_ and NADPH. It is believed to be involved in ascorbate-glutathione (ASC-GSH) cycle (De Gara et al., 2010) and in the biosynthesis of specific defense compounds (e.g., flavonoids, phytoalexins and lignins) (Drincovich et al., 2001). The malic enzyme was abundant at both time points, but exclusively in the susceptible cultivar (spots 2201 and 940, >3 times more). At 6 dpi there is increased abundance of malate dehydrogenase (spot 2701, almost 5 times more), which catalizes conversion of L-malate to oxaloacetate (Journet et al., 1981), which regulates the activity of malic enzyme. The ubiquitin/26S proteasome system can be also involved in antiviral defense pathways probably via degradation of viral movement proteins (Dielen et al., 2010). The 26S proteasome, a multienzyme complex, is involved in degradation of ubiquitinated proteins, protein turnover, and the hypersensitive response (Hanna and Finley, 2007). An increased abundance of proteasome subunit beta, a component of 20S proteasome core, was observed in the partially resistant cultivar Jaguar at 6 dpi (spot 3375, >3 times more) and in Zelena cultivar (spot 2063, almost 2 times more) at disease development stage (15 dpi). Similar response was observed in Kefeng No.1, a resistant soybean cultivar 24 h post-infection by SMV (Yang et al., 2011).

Finally, an increased abundance of actin-7 (spot 2495) at 6 dpi in susceptible cultivar might be related to regulation of cell-to-cell transport of viral particles through stabilization of the cytoskeleton. It is noteworthy that microfilamental actin was also detected in *C. papaya* after *Papaya meleira virus* infection (Rodrigues et al., 2011). It was demonstrated (Su et al., 2010) that *cucumber mosaic virus* and *tobacco mosaic virus* movement proteins inhibited actin polymerization and severed F-actin. The later activity was required to increase the size exclusion limit of plasmodesmata that enable the virus to pass between cells. We speculate that in plants of the susceptible cultivar there is an alternative mechanism to influence viral movement possibly involving stabilization of the cytoskeleton.

## Concluding remarks

**Figure 6:**
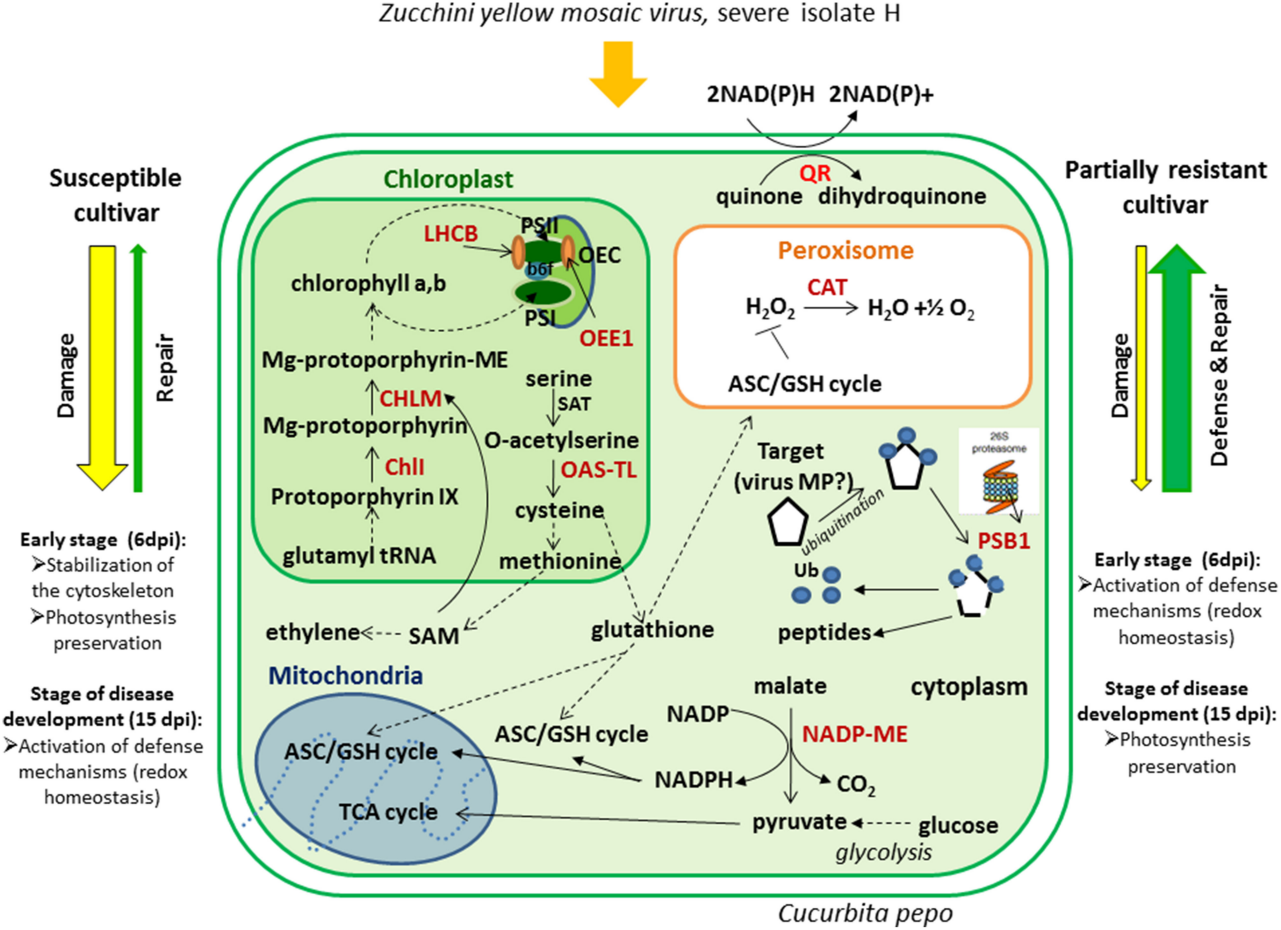
**Cellular responses of *C. pepo* cultivars against ZYMV-H infection**. Catalase (CAT), quinone oxidoreductase (QR), O-acetylserine(thiol)lyase (OAS-TL), NADP-dependent malic enzyme (NADP-ME), Proteasome subunit beta type 1 (PBS1), ubiquitin (Ub), serine acetyltransferase (SAT), virus movement protein (virus MP), S-adenosyl-L-methionine (SAM), magnesium chelatase (ChlI), magnesium-protoporphyrin O-methyltransferase (CHLM), photosystem I (PSI), photosystem II (PSII), light-harvesting chlorophyll a/b-binding proteins (LHCB), oxygen evolving enhancer protein 1 (OEE1), oxygen-evolving complex (OEC), ascorbate/glutathione cycle (ASC/GSH cycle), cytochrome b6f complex (b6f). Some of the identified proteins are highlighted in red.

This study was aimed at clarifying our understanding of the molecular features that underlie the different performance of two *C. pepo* cultivars: Zelena (susceptible) and Jaguar (partially resistant) in response to infection with severe ZYMV-H isolate. Firstly, we have observed clear differences in phenotypical symptoms on leaves due to slower viral development in the partially resistant cultivar in comparison to the susceptible cultivar. Significantly higher viral accumulation was detected by immunoblot analysis in cv. Zelena at 6 dpi. This finding was confirmed by proteomic analyses which revealed an increased abundance of viral proteins in the susceptible cultivar. In order to identify plant proteins that changed in abundance during viral infection, whole cell lysates of both cultivars were resolved by 2-DE at both 6 and 15 dpi. Selected gel spots were studied using LC-MS/MS. ZYMV-H infection in *C. pepo* resulted in an increased abundance of photosynthesis-related proteins in the susceptible cultivar at 6 dpi and at 15 dpi in the partially resistant cultivar. We also observed a rapid activation of proteins involved in defense mechanisms associated with redox homeostasis in the partially resistant cultivar at the early stage of the infection. This was accompanied by lower level of hydrogen peroxide. On the other hand, it appears that the susceptible cultivar is expressing a mechanism to influence a movement of the virus via stabilization of cytoskeleton, plus activating a mechanism to preserve photosynthetic capacity (Figure 6). Summing up, plant immunity is the result of the adjustment of complex metabolic network. While identification of differentially abundant proteins between susceptible and resistant cultivars was an essential prelude, more systematic functional analyses will be necessary to clarify our specific understanding of regulatory pathways involved in plant-pathogen interactions across various cultivars.

### Conflict of interest statement

The authors declare that the research was conducted in the absence of any commercial or financial relationships that could be construed as a potential conflict of interest.

## Supplementary material

Figure S1 **The obtained 2-DE gels of Zelena (S) and Jaguar (R) *C. pepo* cultivars at 6 and 15 dpi with ZYMV**.

Figure S2 **Enlarged view of differentially displayed protein spots that were identified between Zelena (S) and Jaguar (R) *C. pepo* cultivars at 6 dpi and 15 dpi**.

Figure S3 **Column chart of quantitative differences in protein spot intensities observed between Zelena (S) and Jaguar (R) *C. pepo* cultivars in response to infection at both time points (6 and 15 dpi)**. Intensities of the protein spots were calculated with Image Master Platinum software package.

Table S1 **Matching peptides data of proteins identified by LC-MS/MS**.

## Abbreviations

2-DE: two-dimensional gel electrophoresis
dpi: days post-inoculation
LC-MS/MS: liquid chromatography coupled tandem mass spectrometry
ROS: reactive oxygen species
ZYMV-H: *Zucchini yellow mosaic virus* severe isolate H.
