# Supplementary figures and images for: Partially resistant Cucurbita pepo showed late onset of the Zucchini yellow mosaic virus infection due to rapid activation of defense mechanisms as compared to susceptible cultivar
Source: Front Plant Sci. 2015 Apr 28;6:263. doi: 10.3389/fpls.2015.00263 (PMC4411989; doi:10.3389/fpls.2015.00263)

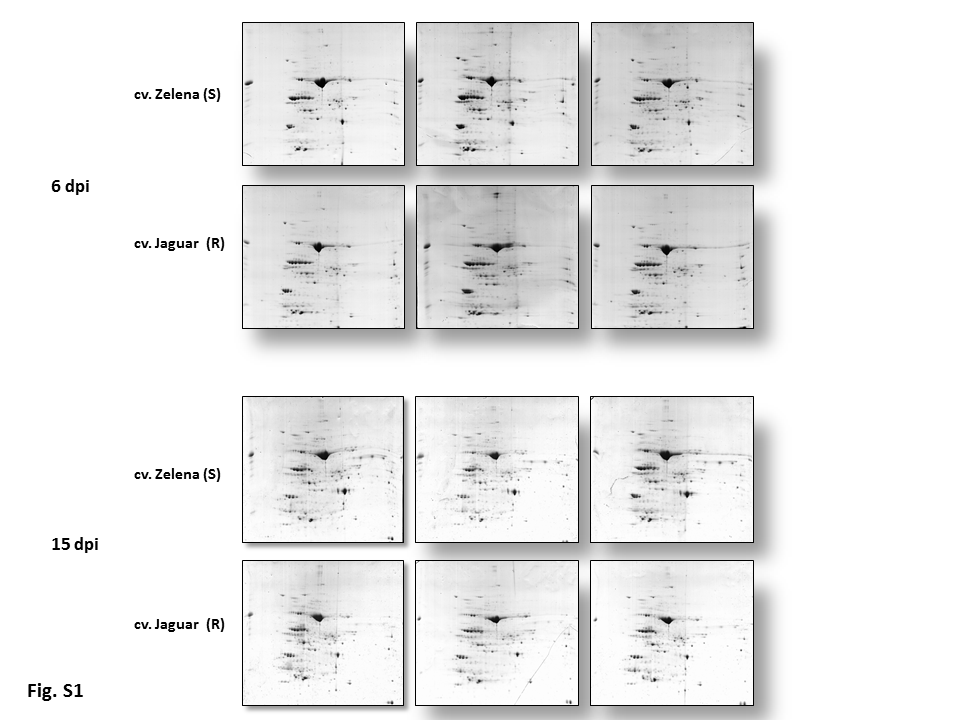

Supplement: Figure S1 — The obtained 2-DE gels of Zelena (S) and Jaguar (R) C. pepo cultivars at 6 and 15 dpi with ZYMV. [file Image1.TIF]

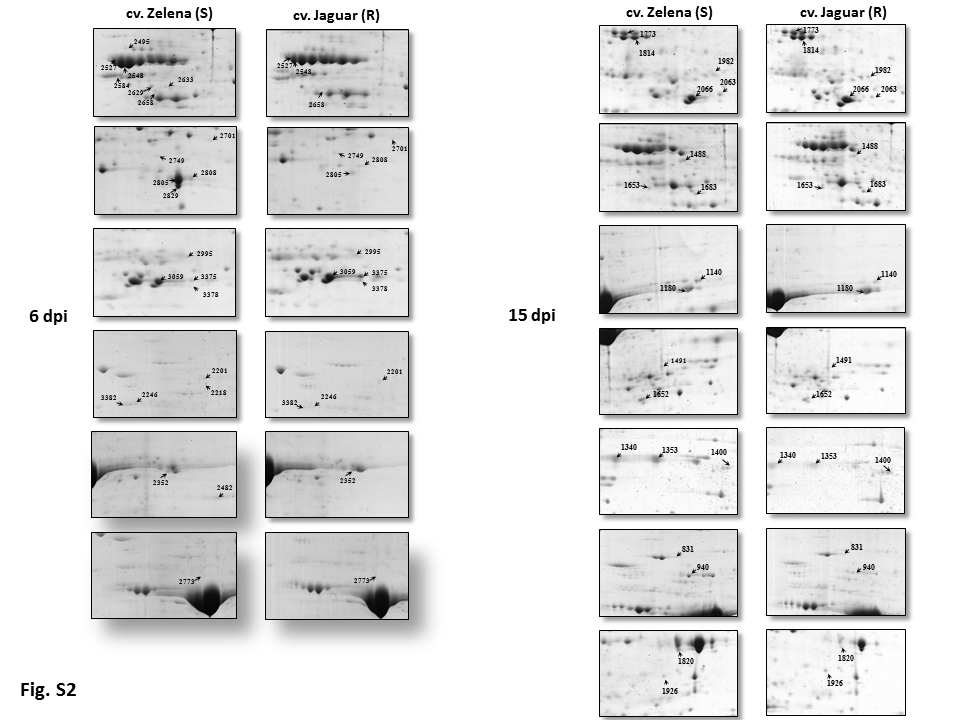

Supplement: Figure S2 — Enlarged view of differentially displayed protein spots that were identified between Zelena (S) and Jaguar (R) C. pepo cultivars at 6 dpi and 15 dpi. [file Image2.TIF]

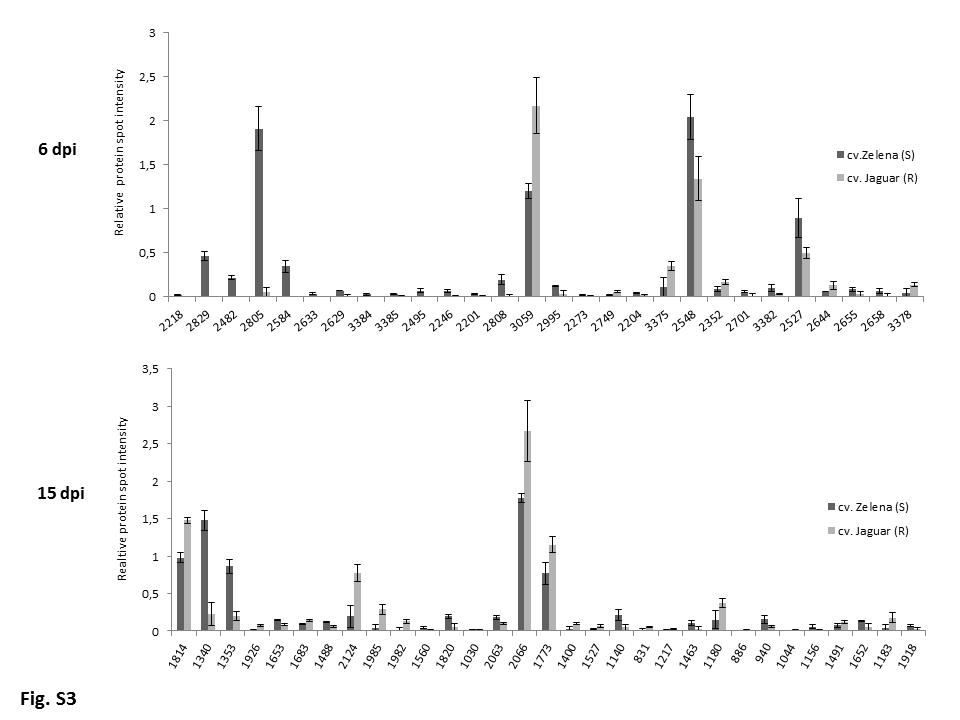

Supplement: Figure S3 — Column chart of quantitative differences in protein spot intensities observed between Zelena (S) and Jaguar (R) C. pepo cultivars in response to infection at both time points (6 and 15 dpi). Intensities of the protein spots were calculated with Image Master Platinum software package. [file Image3.TIF]
